# Beyond inflammation: the multifaceted therapeutic potential of targeting the CXCL8-CXCR1/2 axis in type 1 diabetes

**DOI:** 10.3389/fimmu.2025.1576371

**Published:** 2025-07-11

**Authors:** Georgia Fousteri, Meghan Jones, Rubina Novelli, Serena Boccella, Laura Brandolini, Andrea Aramini, Paolo Pozzilli, Marcello Allegretti

**Affiliations:** ^1^ Research & Development, Dompé Farmaceutici S.p.A, Milan, Italy; ^2^ Research & Development, Dompé US, San Mateo, CA, United States; ^3^ Research & Development, Dompé Farmaceutici S.p.A, Naples, Italy; ^4^ Research & Development, Dompé Farmaceutici S.p.A, L’Aquila, Italy; ^5^ Fondazione Policlinico Universitario Campus Bio-Medico, Rome, Italy; ^6^ Centre of Immunobiology, Blizard Institute, Barts and The London School of Medicine, University of London, London, United Kingdom

**Keywords:** type 1 diabetes, inflammation, chemokines, neutrophils, CXCL8, interleukin-8, CXCR1, CXCR2

## Abstract

Identifying novel therapeutic targets involved in the multiple mechanisms underlying the complex pathophysiology of type 1 diabetes (T1D) could change the natural history of this disease. The CXCL8-CXCR1/2 axis is emerging as a therapeutic target with a crucial, multifaceted role in T1D pathophysiology. CXCL8-dependent neutrophil chemotaxis to the pancreas precedes autoimmunity, and CXCR1/2 blockade mitigates insulitis and T1D development in preclinical models. In parallel, CXCL8 can act in a β cell-autonomous manner, and exert non-immune actions on adipocytes, hepatocytes, podocytes, and muscle cells that contribute to insulin resistance and diabetic complications. In this review, we delineate compelling evidence of immune and non-immune actions of the axis in the onset and progression of T1D. We show that the CXCL8-CXCR1/2 axis represents a promising therapeutic target for the prevention/reversal of T1D, with a meaningful potential clinical advantage conveyed by its role in multiple components of the pathology and diabetic complications.

## 1 Introduction

More than 8 million people globally are diagnosed with type 1 diabetes (T1D), with prevalence expected to reach up to 17.4 million cases by 2040 (1). Insulin therapy has been revolutionary in managing T1D, but is not a definitive cure and does not address the underlying immune-mediated destruction of β cells. The burden of T1D on individuals and healthcare systems worldwide underscores the critical medical need for the development of effective treatments to prevent and cure the disease and delay or prevent secondary complications. However, its complexity and heterogeneity suggest this will require approaches which can target multiple mechanisms and underlying endotypes (1, 2).

The pathogenesis of T1D involves an intricate interplay between environmental factors, the genome, pancreatic β cells and the immune system (3–5), with insulin resistance and secondary complications exacerbating disease progression and patient burden. The CXCL8-CXCR1/2 axis is central to several aspects of T1D pathogenesis and progression and may serve as a common denominator in various endotypes and secondary complications. Here, we describe the crucial roles of the CXCL8-CXCR1/2 axis in T1D onset and progression, with contributions of the axis through immune cells, such as neutrophils and T cells, but also through non-immune cells, such as β cells, adipocytes and podocytes, highlighting its potential as a wide-reaching therapeutic target.

## 2 The CXCL8-CXCR1/2 axis

Chemokines constitute a large family of chemotactic cytokines that exert their action via seven transmembrane G protein-coupled receptors (7TM-GPCRs). The chemokine system is crucial for the regulation and the control of basal homeostatic and inflammatory leukocyte movement (6). CXCL8 (initially interleukin-8 [IL-8]), a member of the CXC chemokine family, is a potent, selective neutrophil chemoattractant produced by macrophages, epithelial cells, and endothelial cells in response to inflammation and infection (6, 7). In addition to cell migration, the CXCL8 axis also controls neutrophil activation and NETosis, a preprogrammed cell death resulting in the release of Neutrophil Extracellular Traps (NETs) which entrap, neutralize, and/or kill pathogenic microorganisms (8–11). Its primary receptors, CXCR1 and CXCR2, are expressed on various cell types in addition to neutrophils, such as monocytes, macrophages, T cells, endothelial cells and certain cancer cells (7).

Elevated CXCL8 levels have been consistently observed in patients with T1D and high CXCL8 levels were shown to correlate with poor glycemic control (i.e., poor hemoglobin A1c [HbA1c] levels) (12–17). Furthermore, neutrophils, the primary target of the axis, have emerged as key players in T1D pathology (3, 18, 19). Below, we delineate compelling evidence of pivotal immune and non-immune actions of the axis in the onset and progression of T1D from the autoimmune trigger through the development of secondary complications.

## 3 Contributions of the CXCL8-CXCR1/2 axis to the pathophysiology of T1D

### 3.1 Susceptibility to T1D via enteroviral infection

Enteroviral infection has been hypothesized to trigger islet autoimmunity either via direct damage to β cells or through bystander activation and molecular mimicry (20–24). CXCL8 has been identified at both the mRNA and protein level in pancreatic islets in response to enteroviral infection, particularly with Coxsackie B virus (CVB) 3, CVB4, or CVB5 (22, 25–27). In one study, CXCL8 was specifically upregulated in islets exposed to a non-lytic CVB strain known to cause persistent infection (VD2921). Such non-lytic strains can cause enduring activation of immune-related genes without any associated cytopathology or cell damage, thereby promoting non-specific inflammation (22). Thus, it may be that virally-induced CXCL8 could mediate immune activation, fueling islet autoimmunity, though its precise role in this context remains ill defined (27).

### 3.2 Endotypes

T1D is a heterogeneous condition characterized by distinct pathophysiological endotypes (28–30). Initially, hyperimmune (young-onset) and pauci-immune (late-onset) endotypes were identified, with increasing complexity leading to proposals of 6 more specific T1D endotypes classified according to factors such as age of disease onset, autoantibody pattern, inflammatory signature, and genetic factors (31, 32). Broadly speaking, the hyperimmune phenotype, more often present in those with disease onset <7 years of age is associated with a higher HLA-conferred genetic risk, greater numbers of autoantibodies at diagnosis, an enhanced interferon- γ (IFNγ) signature, lower C-peptide levels, aberrant proinsulin processing, and higher numbers of pancreatic CD20+ B cells and CD8+ T cells (29–31, 33). In contrast, the pauci-immune signature, often present in those with disease onset ≥13 years of age, is associated with autoantibodies against glutamic acid decarboxylase 65 (GAD-65) (GADA), an enhanced IL-10 signature, more severe metabolic decompensation and associated higher risk of diabetic ketoacidosis (DKA), higher C-peptide levels, and fewer pancreatic CD20+ B cells and CD8+ T cells. Whether neutrophil functions are identified as differentiating features or common drivers of pathology in the these endotypes, will be important to elucidate. Limited available data on the CXCL8-CXCR1/2 axis and neutrophils in the context of endotypes are available thus far and future research should explore whether CXCR1/2 inhibition may be more effective in selected endotypes and at specific disease stages, enabling personalized therapeutic approaches.

Though one study reported that circulating NETosis markers are reduced in T1D patients (34), other reports have shown associations between neutrophil and primary granule genes as well as whole blood neutrophil levels and defining features for both the hyperimmune and pauci-immune phenotypes, including an enhanced type I and type II IFN-related gene signature (hyperimmune) and higher stimulated and fasting C-peptide levels (pauci-immune), in both patients at risk for T1D and T1D patients across disease stages (35, 36). A hybrid phenotype of diabetes, “double diabetes” (DD), has also emerged which is characterized by canonical symptoms of both T1D and type 2 diabetes (T2D), that is, both autoantibody positivity and obesity-related insulin resistance (30, 37, 38). Notably, the diversity of mechanisms driving T1D progression across different endotypes and the resulting inter-subject variability may necessitate the development of a panel of disease-modifying therapeutic options for patients, within which CXCR1/2-tarteging therapies may be an important player (32). The hypothesis that CXCL8 may serve as a common denominator across endotypes, including DD, is supported by the significant contributions of the CXCL8-CXCR1/2 axis to insulin resistance as outlined below.

### 3.3 Immune actions of the CXCL8-CXCR1/2 axis: neutrophil proinflammatory actions

#### 3.3.1 Autoimmunity

**Figure 1:**
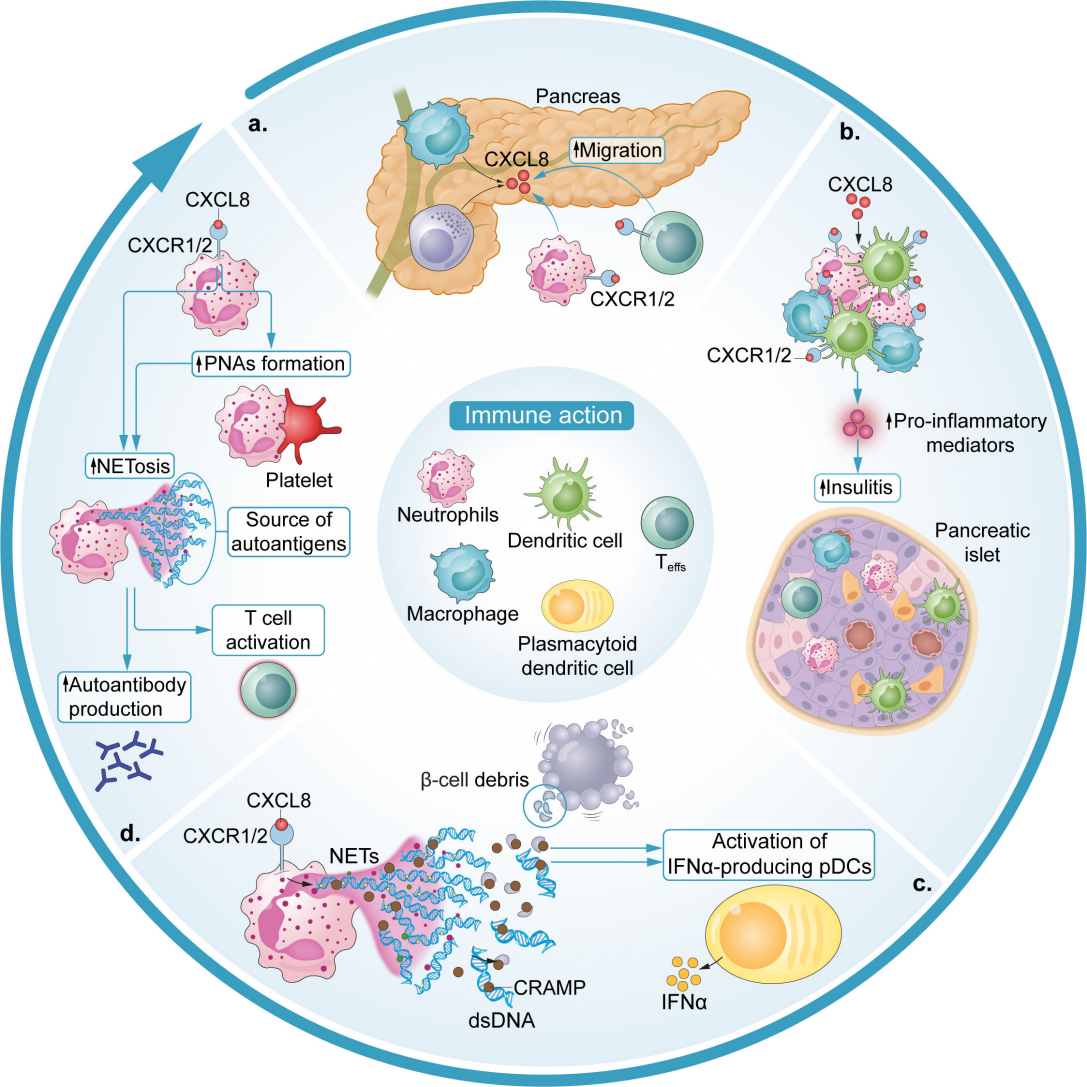
Immune actions of the CXCL8-CXCR1/2 axis in T1D. **(a)** Macrophage- and β cell-derived CXCL8 induces neutrophil and possibly autoreactive T cell chemotaxis to the pancreas. **(b)** CXCL8 initiates a positive feedback loop, mostly propagated by recruited neutrophils and macrophages, which exacerbates and amplifies insulitis with the release of additional proinflammatory mediators. **(c)** Neutrophil NETosis activates IFNα-producing pDCs. Specifically, the release of NET-derived CRAMP complexes with β cell debris and dsDNA (self-DNA) and activates IFNα-producing pDCs which serve as antigen-presenting cells. **(d)** PNAs contribute to a positive feedback loop of platelet – neutrophil activation, which is linked to NETosis and, subsequently, promotes autoantibody production via NETosis ultimately leading to T cell activation.

Though it is well-established that T1D is a T cell-mediated disease whereby islet-specific, autoreactive CD4+ and CD8+ effector T cells (Teffs) drive β cell destruction (39–41), T cells do not trigger autoimmunity in isolation (**Figure 1**). Neutrophil activation and migration to the pancreas have been associated with the presence of autoantibodies and identified as a critical step in T1D pathogenesis (3, 6, 18, 19, 42). In T1D patients, a higher degree of pancreatic neutrophil infiltration has been associated with poor β cell function, even in presymptomatic individuals before T1D diagnosis (35). Preclinical studies have suggested that neutrophil migration in the pancreas in the initial stages of islet inflammation is driven by macrophage and β cell-derived CXCL8 and CXCL2 (3, 18, 42). *In vitro*, dose-dependent neutrophil chemotaxis was observed in response to recombinant CXCL8 in a trans-well migration assay (42). Neutrophil chemotaxis in the same assay was also observed in response to conditioned medium containing high levels of CXCL8 produced by stressed EndoCβ-H1 cells (a human β cell line) and this was prevented by the application of a CXCL8 neutralizing antibody (42). *In vivo*, upregulations in circulating macrophage CXCL1 expression, a homologue of CXCL8, were observed in spontaneously diabetic biobreeding (BB) rats and in a streptozotocin (STZ) -induced diabetes mouse model (3, 43, 44). Pancreatic neutrophil CXCR2 expression was also upregulated in the non-obese diabetic (NOD) mouse model and CXCR2 blockade via the CXCR2 antagonist SB225002 prevented both the pancreatic neutrophil infiltration normally occurring in NOD mice and the rapid influx of neutrophils to the pancreas induced by STZ (18, 45). Importantly, CXCR2 blockade at 8 weeks of age in NOD mice was sufficient to significantly reduce the presence of islet autoreactive CD8+ T cells and led to a reduction in the prevalence of T1D in mice up to 40 weeks of age (3).

The protective effect of CXCR2 blockade on diabetes development was replicated with two additional blockers. Ladarixin, an allosteric non-competitive CXCR1 and CXCR2 antagonist, delayed the onset of diabetes and improved glycemic control when administered following multiple low-dose STZ injections in C57BL/6 mice (45). Similarly, ladarixin treatment in 12-week old NOD mice delayed and prevented the onset of spontaneous diabetes and the severity of insulitis, with only 22% of ladarixin-treated mice developing diabetes during follow up relative to 78% of vehicle-treated mice. This was accompanied by a reduction in numbers of intrapancreatic neutrophils, macrophages and lymphoid cells, and a reduction in their associated CXCR2 expression. Strikingly, ladarixin treatment in NOD mice with recent-onset diabetes induced a rapid reversal of diabetes (45), strongly suggesting that the CXCL8-CXCR1/2 axis makes important contributions to T1D progression. Reparixin, another CXCR1/2 antagonist, was assessed in the context of islet transplantation outcomes and also improved islet engraftment in C57BL/6 mice (46).

Compelling evidence for a significant role of the CXCL8-CXCR1/2 axis in T1D gleaned from *in vivo* studies of CXCR1/2 blockade in preclinical models is underscored by further discoveries about neutrophil actions in this context. Neutrophil activation characteristic of NETosis and inflammation (e.g., neutrophil elastase (NE), myeloperoxidase (MPO), proteinase-3 (PR-3), protein arginine deiminase 4 (PAD4), LL37 and cell-free DNA-histone complexes, and IFN-responsive genes) has been observed in diabetic mice and recent-onset T1D patients (3, 17, 35, 47, 48), with levels of MPO remaining significantly increased relative to healthy controls even in long-term T1D patients (47, 49). Platelet-neutrophil aggregates (PNAs) have also been shown to increase in circulation ahead of T1D onset, initiating a positive feedback loop of platelet-neutrophil activation which has been linked to autoantibody positivity and recent-onset T1D (19). Further, NETs have been suggested as a direct source of autoantigen in various autoimmune diseases (i.e., anti-citrullinated protein antibodies in Rheumatoid arthritis [RA]). It is hypothesized that they may contribute to post-translationally modified islet autoantigens in T1D (50–54). While therapeutic approaches to inhibit NETosis have been considered for SLE, RA, and small-vessel vasculitis, NETosis inhibitors are not currently in development for the treatment of T1D (55).

Diana and colleagues reported the presence of NETs and associated release of DNA-binding cathelicidin-related antimicrobial peptide (CRAMP) in the pancreatic islets of NOD mice at 3 weeks of age (3). Subsequent experiments demonstrated that interactions between NET-derived CRAMP and complexes of β cell debris (i.e., self-DNA) and dsDNA-specific immunoglobulin G (IgG) secreted by local B1a cells cooperatively activated IFNα-producing, autoantigen-presenting plasmacytoid dendritic cells (pDCs) (3, 56). NETosis was confirmed to be a critical component to this pDC activation in that neutrophil depletion was sufficient to prevent the induction of IFNα secretion from islet pDCs. Both IFNα-secreting pDCs and the diabetogenic T cell response in the islets of 8-week old NOD mice were mitigated by neutrophil depletion (3).

Possibly, approaches targeting CXCR1 and CXCR2 do not affect solely neutrophils, as both receptors are expressed on terminally differentiated Teffs (57–60). Upregulation of CXCR1/2 on islet-specific T cells may enhance their migration to the pancreas. One study showed that approximately 25% of intrahepatic NKT cells expressed CXCR2 in C57BL/6 mice (46). Intrahepatic NKT levels increased markedly upon islet transplantation, but this was mitigated with reparixin treatment (45). Outside the context of T1D, CXCR1 expression on T cells has been associated with enhanced IFNγ expression, enhanced cytotoxicity, pro-apoptotic factors and higher levels of death-associated protein kinase 1 (DAPK1) (58–60). This suggests that higher expression of CXCR1 on CD8+ T cells may render them prone to enhanced cytotoxicity. Lastly, enhanced T cell CXCR1 expression has been reported in other autoimmune diseases, for example, on CD3+ T cells in RA (61) and inflammatory bowel disease (62) and on Vδ2 T cells in SLE (63).

T cells are one of the most common targets of NET contents (64). Direct contact with NETs has been shown to prime T cells by lowering T cell activation thresholds (65). Furthermore, activated neutrophils were shown to induce Th1 or Th17 polarization via release of IL-12 or Th17-specific transcription factors (66, 67) or by guiding the cytokine profile or expression of costimulatory molecules (e.g., CD80, CD86, or HLA-DR) in DCs (67–69). Evidence has also suggested that neutrophils contain reserves of costimulatory molecules themselves such that, under conditions of inflammation, their expression can be translocated to the surface and neutrophils can present antigens to T cells directly (70–72).

Finally, intricate neutrophil-regulatory T cell (Treg) interactions play a key role in islet-specific self-tolerance. Tregs, which critically suppress autoreactive Teffs to maintain a balance of immune activity under healthy conditions (73, 74), have been shown to exhibit considerable impairments in their ability to constrain excessive autoimmune actions and maintain self-tolerance in T1D (75, 76). Specifically, Tregs collected from patients with T1D exhibited a weakened ability to limit proliferation of autologous Teffs and a shifted profile of cytokine secretion favoring more proinflammatory cytokines and fewer anti-inflammatory cytokines relative to those collected from healthy controls (73, 75–78). Interestingly, while Tregs are traditionally known for releasing anti-inflammatory signals, i.e., IL-10 and transforming growth factor-β (TGF-β), recent discoveries have indicated that CD4+ FOXP3+ Tregs can also secrete CXCL8, employing CXCL8-driven chemoattraction to recruit neutrophils (79). By attracting neutrophils to their vicinity, Tregs could induce the expression of anti-inflammatory markers, including suppressor of cytokine signaling 3 (SOCS3), IL-10, and TGF-β, promoting neutrophil apoptosis (80–82). This reveals an additional mechanism through which Tregs contribute to autoimmunity suppression, i.e., by imparting tolerogenic signaling to neutrophils. In line with this, defective Treg function and concurrent overactivation of neutrophils have been observed in autoimmune diseases like SLE, vasculitis, and RA, indicating a potential link between Treg dysfunction and neutrophil overactivity (53, 82–87). It remains unclear whether this mechanism is operational in T1D.

#### 3.3.2 Insulin resistance (immune actions)

Insulin resistance, the inability of cells to respond effectively to insulin, is commonly associated with T2D. However, it also occurs in some individuals with T1D, especially in those with obesity and during puberty and pregnancy (88–91) and encompasses what is now considered the DD endotype (30, 37, 92). Chronic inflammation is a known component of insulin resistance in obese and diabetic patients (93). In obesity, adipose tissue can release proinflammatory cytokines (e.g., TNF, IL-6), CXCL8, and adipokines (e.g., leptin), dysregulating leukocyte trafficking (94). Under conditions of hyperglycemia, abnormal leukocyte trafficking driven by inflamed adipocyte extracellular vesicles contributes to enhanced leukocyte-endothelial cell adhesion, increased production of advanced glycosilation end products (AGEs), generation of reactive oxygen species (ROS) and increased expression of cell adhesion molecules (CAMs). Talukdar and colleagues showed that mice exposed to a high-fat diet (HFD) exhibited an increase in adipose tissue neutrophil levels and markers of NETosis (i.e., NE) which emerged within 3 days of HFD exposure and persisted through 90 days (95). The administration of an NE inhibitor, GW311616A, improved glucose tolerance, an indicator of insulin resistance, in HFD-fed mice, while application of recombinant mouse NE led to a substantial increase in glucose intolerance. Deletion of NE in mice also protected against the development of insulin resistance, with a 90% reduction in adipose tissue-infiltrating neutrophils compared to wild-type mice (95).

Human and animal studies have shown CXCR1/2 agonists (e.g., CXCL8, CXCL5 in T1D patients, CXCL1 in mice) to be correlated with obesity (12–16) and insulin resistance (96, 97). Both genetic deletion of CXCR2 (96, 97) and CXCR1/2 blockade in HFD-fed mice and db/db mice protected against insulin resistance (98, 99). CXCR2 knockout or CXCR2 blockade led to stronger protective effects than did a CXCL5-neutralizing antibody (96), another chemokine which binds CXCR2, and additionally influenced macrophage polarization (i.e., shifted the balance between proinflammatory M1 and anti-inflammatory M2 macrophages) in db/db mice (98).

### 3.4 Non-immune actions of the CXCL8-CXCR1/2 axis

#### 3.4.1 ER stress

**Figure 2:**
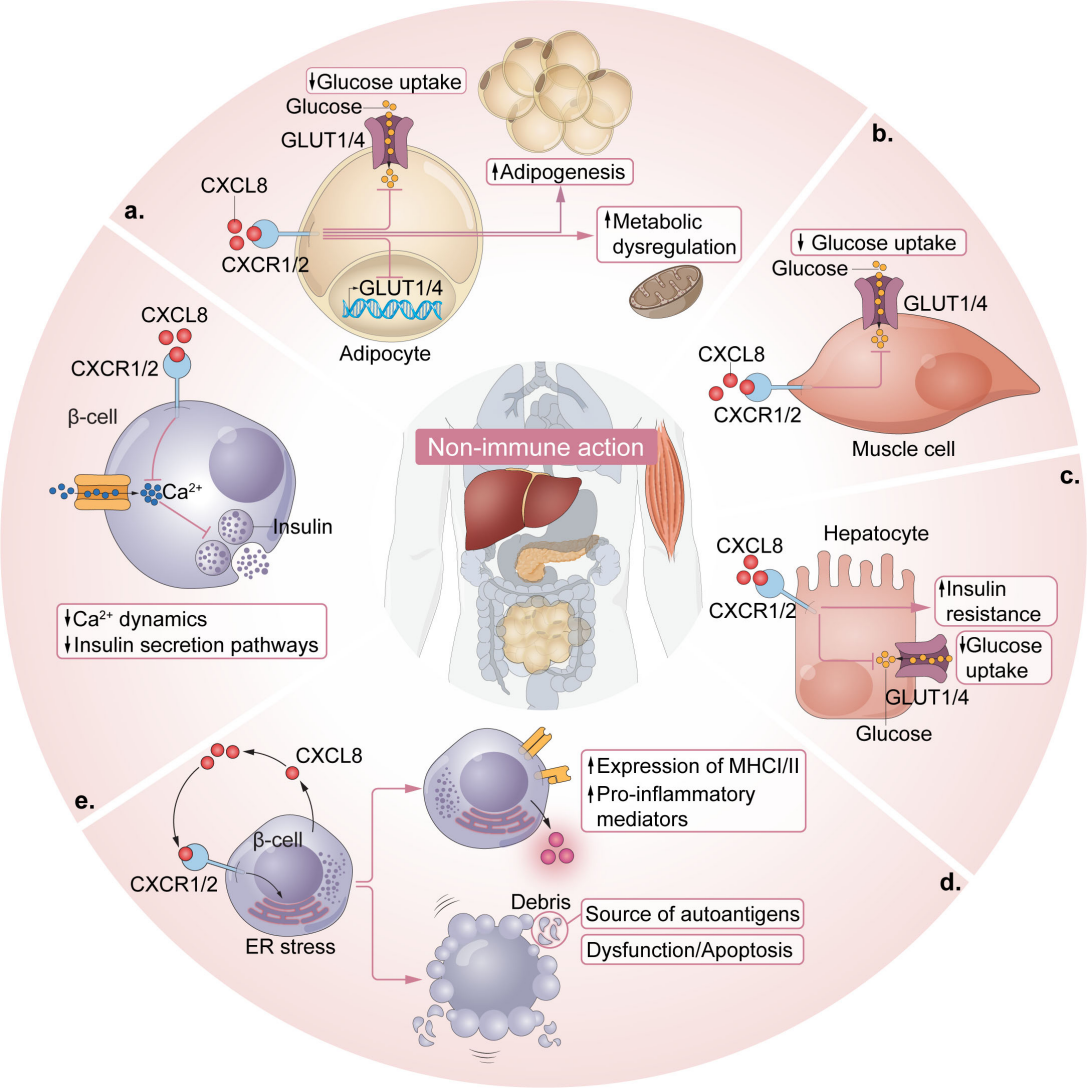
Non-immune actions of the CXCL8-CXCR1/2 axis in T1D. **(a)** Adipocyte CXCR1/2 signaling reduces glucose transport and disrupts metabolism by downregulating the surface expression of glucose transporters, GLUT4 and GLUT1. Adipocyte CXCR2 activation can drive adipogenesis, further amplifying these effects. **(b)** Muscle cell CXCR2 receptors influence insulin-stimulated glucose transport, with CXCL5-CXCR2 activation shown to reduce glucose transport. **(c)** Hepatocyte CXCR1/2 activation facilitates inflammation-induced insulin resistance and inflammation-induced reductions in GLUT transporters/glucose transport on hepatocytes. **(d)** β cell ER stress is sufficient to induce β cell dysfunction, apoptosis, overexpression of MHCI/II and the release of proinflammatory mediators, including CXCL8, which facilitate further β cell ER stress and promote islet-specific autoimmunity. Importantly, β cell debris can serve as autoantigen material. **(e)** CXCL1- and CXCL5- activation of CXCR2 impairs β cell function (e.g., insulin secretion pathways) by affecting intracellular calcium dynamics in pancreatic islets.

Recent converging evidence supports a direct role of β cells in their own demise in that intrinsic β cell impairment or death may trigger a cascade of events that contribute to autoimmunity in T1D. Endoplasmic reticulum (ER) stress can drive β cell dysfunction, apoptosis, overexpression of MHC class I/II, and release of proinflammatory cytokines and chemokines, while β cell debris serve as autoantigens (**Figure 2**) (3, 42, 100–104).

β cell ER stress has been shown to engage CXCL8-CXCR1/2 axis signaling in human β cells *in vitro* and in muscle insulin-resistant zebrafish (zMIR) (42, 104, 105). Thapsigargin-induced ER stress in EndoCβ-H1 cells (a human β cell line) produced a type 1 IFN response and dsDNA leakage along with a strong induction of CXCL8 and CXCL8-dependent neutrophil chemotaxis (42). In zMIR, ER stress induced by overnutrition initiated macrophage recruitment and TNF release along with subsequent CXCL8 release from β cells (104). ER stress-induced β cell loss was associated with “hot spots” of neutrophil chemotaxis and genetic deletion of CXCL8 prevented ER stress-induced β cell death (104). Thus, CXCL8 may act in a β cell-autonomous manner, contributing to the consequences of ER stress and facilitating ER stress-induced β cell death within the islets.

#### 3.4.2 Insulin resistance (non-immune actions)

CXCR1/2 signaling makes substantial contributions to insulin resistance directly via non-immune cells like adipocytes, muscle cells, and β cells (**Figure 2**) (96, 106, 107). In one study, the molecular mechanisms underlying the antidiabetic effect of CXCR1/2 blockade were evaluated in differentiated adipocytes (106). Hyperglycemic or inflammatory conditions (i.e., CXCL1), or the combination of both, upregulated CXCR1/2 expression on adipocytes and reduced glucose transport *in vitro* (106). CXCR1/2 blockade mitigated CXCR1/2 expression in adipocytes under hyperglycemic conditions and restored glucose uptake and glucose transporter levels (GLUT4 and GLUT1) to control levels. It also reduced lipolysis and cytokine release while enhancing adiponectin levels, suggesting metabolic restoration. In human adipocytes from an obese patient, blockade of CXCR1/2 reduced the size of adipocytes and reduced the release of CXCL8 and other inflammatory cytokines and angiogenic factors (106). These data are consistent with those from two other studies which demonstrated that application of CXCL1 to cultured adipocytes enhanced inflammation, inducing release of leptin, monocyte chemotactic protein-1 (MCP-1), IL-6, and TNF, as well as SOCS3, a factor known to mediate insulin resistance (97, 108).

Dyer and colleagues reported that female cxcr2 null mice displayed a unique adipocyte phenotype, characterized by small and fewer adipocytes constituting a thin adipose layer, and a reduced expression of adipogenesis-related genes, peroxisome proliferator-activated receptor (PPARγ) and fatty acid binding protein 4 (FABP4), in fat depots (107). Consistent with this, adipocytes exhibited reduced differentiation in the presence of a CXCR2 inhibitor. These data suggest a direct role for adipocyte-specific CXCR2 signaling in adipogenesis, which could have important implications for insulin resistance (107).

Studies of obesity-related insulin resistance in preclinical models have shown direct effects of the CXCL8-CXCR1/2 axis on muscle cells and β cells. In soleus muscle tissue from obese db/db mice, CXCL5 inhibited insulin-stimulated glucose transport by inhibiting the phosphorylation of Akt and activation of SOCS2 via the JAK/STAT pathway (96). This effect was mitigated by CXCR2 blockade. With regard to β cells, *in vitro* application of CXCL5 and CXCL1 was shown to influence β cell intracellular calcium dynamics, which are indicative of insulin secretion capabilities, in pancreatic islets (109, 110).

Finally, the liver is a key insulin-sensitive organ which influences glucose homeostasis (111). Insulin resistance in hepatocytes directly contributes to glucose metabolism, hyperglycemia and glucose intolerance (112). Previous studies have demonstrated that hepatocytes express CXCR1 and CXCR2 receptors (113, 114) and insulin signaling dynamics in hepatic cells were sensitive to TNF both *in vitro* and *in vivo* (115, 116). A recent study assessed the effects of CXCR1/2 blockade on inflammation-induced insulin resistance in hepatocytes *in vitro* (117). TNF-induced insulin resistance in hepatocytes was mitigated by CXCR1/2 blockade through the inhibition of intracellular inflammatory pathways such as JNK and NF-κB and positive modulation of the metabolic profile of the cells. Similar to the results obtained in adipocytes, CXCR1/2 blockade restored glucose uptake and GLUT protein levels and reestablished insulin sensitivity in hepatocytes.

## 4 Contributions of the CXCL8-CXCR1/2 axis in T1D-associated secondary complications

Patients with T1D often face various secondary complications affecting multiple organ systems. Abnormalities in the CXCL8-CXCR1/2 axis exist at a multi-organ level and may contribute to the pathophysiology of some of these complications.

### 4.1 Cardiovascular complications

Not surprisingly, obesity and insulin resistance, which are becoming increasingly common components of T1D and DD in particular, are strongly linked to cardiovascular disease (118, 119). Two recent studies reported enhanced endothelial cell-derived CXCL8 in patients with T1D in key cell types involved in atherogenesis/atherosclerosis. Li and colleagues showed that coronary artery endothelial cells derived from patients with T1D secreted elevated levels of CXCL8, despite comparable toll-like receptor (TLR) 2 and TLR4 levels; and this effect was not reversed by application of exogenous insulin (120). Similarly, hyperglycemic conditions were sufficient to induce CXCL8 secretion in macrovascular aortic endothelial cells *in vitro* (121).

Improvements in markers for risk for cardiovascular disease (low-density lipoprotein [LDL]- and high-density lipoprotein [HDL]- cholesterol levels) in patients with T1D were associated with a reduction in LPS-induced secretion of CXCL8 from monocytes (122). Consistent with this, LDL from T1D patients enhanced CXCL8 and MCP1 expression in endothelial cells *in vitro*, irrespective of glycemic status (123). Of note, markers of NETosis have also been reported to promote poor cardiac outcomes in non-diabetic patients (124, 125).

### 4.2 Diabetic ketoacidosis

DKA was associated with an increase in plasma CXCL1 and CXCL8 in pediatric T1D patients (126) and an increase in blood CXCL1 in STZ-induced diabetic mice was associated with ketoacidosis (127). *In vitro*, stimulation of microvascular endothelial cells with plasma collected from T1D patients with DKA led to enhanced neutrophil - endothelial cell adhesion, and this was prevented by the application of antibodies against CXCL1/8 or CXCR1/2 blockers. Similarly, monocyte and endothelial cell CXCL8 secretion was induced *in vitro* by the application of a hyperketonemic state (128).

### 4.3 Nephropathy

Diabetic nephropathy is the most common cause globally of renal failure (129, 130). In both T1D and T2D, urinary CXCL8 has been shown to be elevated in patients with diabetic nephropathy (relative to diabetic patients without nephropathy) and related to poor renal function (i.e., glomerular filtration rate, microalbuminuria, progression vs. stability of renal decline, albumin:creatinine ratio, clinical outcome) (131–134). Ambinathan and colleagues published a thorough description of the relationship between renal function and urinary and serum inflammatory markers, including CXCL8, in a large cohort of patients with diabetic nephropathy (131). In their study, urinary CXCL8 predicted reduced renal efferent arteriolar function and was significantly increased in urine following an infusion of angiotensin II (131).

CXCL8 and its receptors have been detected in human and murine glomeruli, and in immortalized human podocyte cells (133). *In vitro*, podocyte CXCL8 release was strongly enhanced during a high-glucose challenge, suggesting that hyperglycemia may trigger podocyte CXCL8 production in diabetic patients, which could in turn activate death signals through an autocrine loop and facilitate diabetic nephropathy (133). Similar results were observed in experiments investigating glucose-dependent CXCL8 signaling in podocytes obtained from differentiation of renal stem cells (RSC) cultured as nephrospheres (135, 136). In RSC-differentiated podocytes, a high-glucose challenge induced an increase of CXCL8 transcript expression and protein secretion and DNA damage, and these effects were prevented by CXCR1/2 blockade (i.e., application of ladarixin) (136). Moreover, upon incubation on healthy leukocytes, supernatant from high-glucose challenged podocytes, but not high-glucose challenged epithelial cells, nominally enhanced leukocyte-mediated secretion of proinflammatory cytokines, suggesting that crosstalk between immune and non-immune cells could contribute to the progression of diabetic nephropathy. CXCR1/2 blockade via ladarixin mitigated this podocyte-dependent increase in leukocyte secretion of proinflammatory cytokines (136). *In vivo*, CXCR1/2 blockade reduced albuminuria, mesangial expansion, and podocyte apoptosis and DNA damage in db/db mice (133). Thus, though a few studies reported no relationship between urine/serum CXCL8 and renal function in patients with T1D and microalbuminuria (137–140), there is substantial evidence warranting further exploration of the potential clinical benefit of CXCR1/2 inhibition in diabetic nephropathy.

### 4.4 Diabetic neuropathy and retinopathy

While not many studies have explored a potential role for the axis in diabetic neuropathy, serum CXCL8 in patients with T1D was significantly higher in patients with diabetic neuropathic pain relative to diabetic controls (141) and was correlated with cold perception threshold in patients with childhood-onset T1D (142). Ocular CXCL8 has also been shown to be elevated in patients with diabetic retinopathy relative to nondiabetic controls and was related to measures of disease severity and disease progression in several studies (143–146). A recent experiment evaluating STZ-induced signs of diabetic complications in rat employed a unique design which separated protective effects of CXCR1/2 blockade potentially secondary to its effect on β cell function and glycemic control from protective effects of CXCR1/2 blockade due to direct actions on the underlying pathophysiology of neuropathy or retinopathy (147). Specifically, late repeated ladarixin treatment, i.e., beginning 8 weeks post-STZ when β cell loss and dysglycemia were resistant to treatment, was still sufficient to mitigate STZ-induced signs of diabetic peripheral neuropathy (e.g., mechanical allodynia and thermal hyperalgesia) and diabetic retinopathy (e.g., vitreous and retinal inflammatory [CXCL1, CXCR1/2, myeloperoxidase, citrullinated histone H3] and pro-angiogenic (vascular endothelial growth factor, CD34) factors. This suggests a direct role of the CXCL8-CXCR1/2 axis in diabetic peripheral neuropathy and retinopathy.

## 5 CXCR1/2 as a therapeutic target in T1D: unanswered questions

### 5.1 Current and emerging T1D therapies

Identifying patients at prodromal stages of T1D offers a crucial therapeutic window for preventing disease progression and symptom onset (148, 149). Meanwhile, individuals with late-stage or long-standing T1D require treatments targeting acute symptoms and secondary complications. Current prevention strategies focus on preserving β cell mass by intercepting autoimmunity, whereas in longstanding disease marked by significant β cell loss, therapeutic strategies prioritize restoring β cell function through replacement or regeneration while also preventing autoimmunity recurrence.

While technological advances such as continuous glucose monitoring devices and/or insulin pumps have improved quality of life for some patients (150, 151), the use of external insulin carries a dual burden, being both costly and entailing significant risks due to potential complications like severe hypoglycemia or hyperglycemia. An artificial pancreas, or closed loop insulin system involving continuous glucose monitoring and an implanted insulin pump, has been approved for use in T1D (152, 153), but its use is also limited by its high cost and a lack of education around the device.

Current and emerging disease-modifying therapies that aim to preserve or restore β cell function rather than simply mitigate symptoms of T1D rely on immune-focused approaches, with varying degrees of success. These include cell-based therapies or islet transplantation, agents targeting Teffs and B cells, as well as nonspecific anti-inflammatory agents against TNF, IL-1β, IL-21 or IL-6 (41). For example, rituximab, an anti-CD20 antibody which depletes B cells, was associated with significant but only transient preservation of β cell function in new-onset T1D patients (154, 155). Similarly, the role of cytokines such as TNF, IL-1β, or IL-6 in innate immunity suggest they could represent targets with key roles in triggering autoimmunity. While the TNF-targeting agents etanercept and golimumab significantly improved HbA1c levels and endogenous insulin in children with new-onset T1D (156) and improved C-peptide levels in children and young adults with newly diagnosed (overt) stage 3 T1D (157), the IL-1β-targeting agents anakinra or canakinumab, and the anti-IL-6 antibody, toclizumab, failed to show improvements in recent-onset T1D patients (158, 159). It is possible that a more wide-reaching anti-inflammatory approach, such as the use of JAK inhibitors, would convey advantages over these agents. Indeed, baricitinib was shown to improve β cell function in a recent pilot trial (160). Notably, combination therapies represent an intriguing approach to improve outcomes. For example, combination therapy with an anti-IL-21 agent to target CD8+ Teff, Th17 and Th cells and the glucagon-like peptide-1 receptor agonist (GLP-1RA) liraglutide preserved β cell function in recent-onset T1D patients in a Phase 2 trial with a stronger safety profile than immunosuppressive, disease-modifying therapies alone (161). As another example, reparixin in combination with islet transplantation has been considered (46, 162).

Substantial variability in response to candidate T1D therapies in clinical trials complicates the clinical landscape for T1D treatment, as response to treatment may vary based on stage of disease, disease severity or the underlying endotype. For example, T cell- and B cell-targeting therapies have shown some efficacy in improving outcomes specifically for stage 3 (teplizumab, abatacept, rituximab) (154, 163–165) and stage 2 (teplizumab) (166) diabetes. Among symptomatic patients, disease severity (e.g., baseline β cell function) may determine response to some treatments. An improvement in C-peptide levels following teplizumab treatment has been associated specifically with stage 2 T1D with low β cell function at baseline and with the specific HLA genotypes HLA-DR3- negative or HLA-DR4- positive (166, 167). Similarly, patients with the hyperimmune endotype associated with stronger B cell and T cell infiltration may be expected to respond more favorably to immune-targeting therapies (i.e., rituximab, teplizumab), relative to those with the pauci-immune phenotype (30, 33, 154). Accordingly, future clinical trials should consider this heterogeneity in their designs and plan for population-specific analyses/evaluations. 5.2 CXCL8-CXCR1/2 axis-targeting therapies

Based on this existing clinical landscape, it is clear that there is still a substantial unmet need for disease-modifying therapies for T1D across disease severity/progression and heterogenous patient populations. What emerges from a thorough analysis of the literature available on the CXCL8-CXCR1/2 axis in the context of T1D is that this signaling pathway is central to a complex interplay between immune and non-immune cells contributing to β-cell dysfunction and insulin resistance, as well as secondary complications, making the axis particularly well positioned to serve as a therapeutic target which could convey advantages over other agents discussed. Several compounds targeting this axis have been studied so far. SB220052, a selective CXCR2 inhibitor, and reparixin and ladarixin have shown promise to prevent the development of T1D in preclinical studies (18, 45). In patients, a Phase 2 open-label pilot study revealed that reparixin treatment improved islet transplantation success, as indicated by improved glycemic control, decreased insulin requirement, and appearance of detectable levels of C-peptide well above 0.3 ng/ml (46), however, this was not replicated in a later Phase 3 trial (162). Although a randomized, double-blind, placebo-controlled Phase 2 trial (NCT02814838) in recent-onset T1D with ladarixin failed to achieve its primary endpoint, improvement in C-peptide response to a mixed-meal tolerance test, a significant fraction of ladarixin-treated patients in this trial achieved <7% HbA1c levels without severe hypoglycemic events (SHE) (168). Moreover, improved C-peptide were observed in a subgroup of patients with low fasting C-peptide levels at baseline (multiple metrics) (168) and a predefined subgroup analysis of the efficacy of ladarixin stratified by baseline daily insulin requirement revealed a significant improvement in C-peptide levels in ladarixin-treated patients with high daily insulin requirements (169).

Importantly, CXCR1/2 inhibitors have been well tolerated in patients with T1D in studies thus far, with no clinically relevant safety observations detected (162, 168). However, the broad expression and pleiotropic functions of CXCL8 and its receptors raise concerns about potential off-target effects, particularly with chronic use. Safety will also be critical to assess in vulnerable subpopulations such as children or individuals with immunocompromising comorbidities or other comorbid autoimmune diseases. Long-term safety data from other indications should be cautiously extrapolated to T1D, and dedicated studies are needed to assess immune competence, metabolic impact, and safety profiles across different age groups and disease stages.

Based on these promising results, further trials of therapies targeting this axis are strongly warranted. Currently, two ongoing double-blind, placebo-controlled studies are examining the efficacy of ladarixin in patients with recent-onset T1D, a Phase 2 study (NCT04899271) to evaluate ladarixin in patients with preserved β cell function and a Phase 3 study (NCT04628481) to evaluate ladarixin in patients with low residual β cell function. In parallel, a Phase 2 trial (2020-003296-18) is ongoing to investigate the effect of ladarixin on insulin sensitivity in obese prediabetic patients eligible to receive bariatric surgery.

### 5.2 Future directions

**Figure 3:**
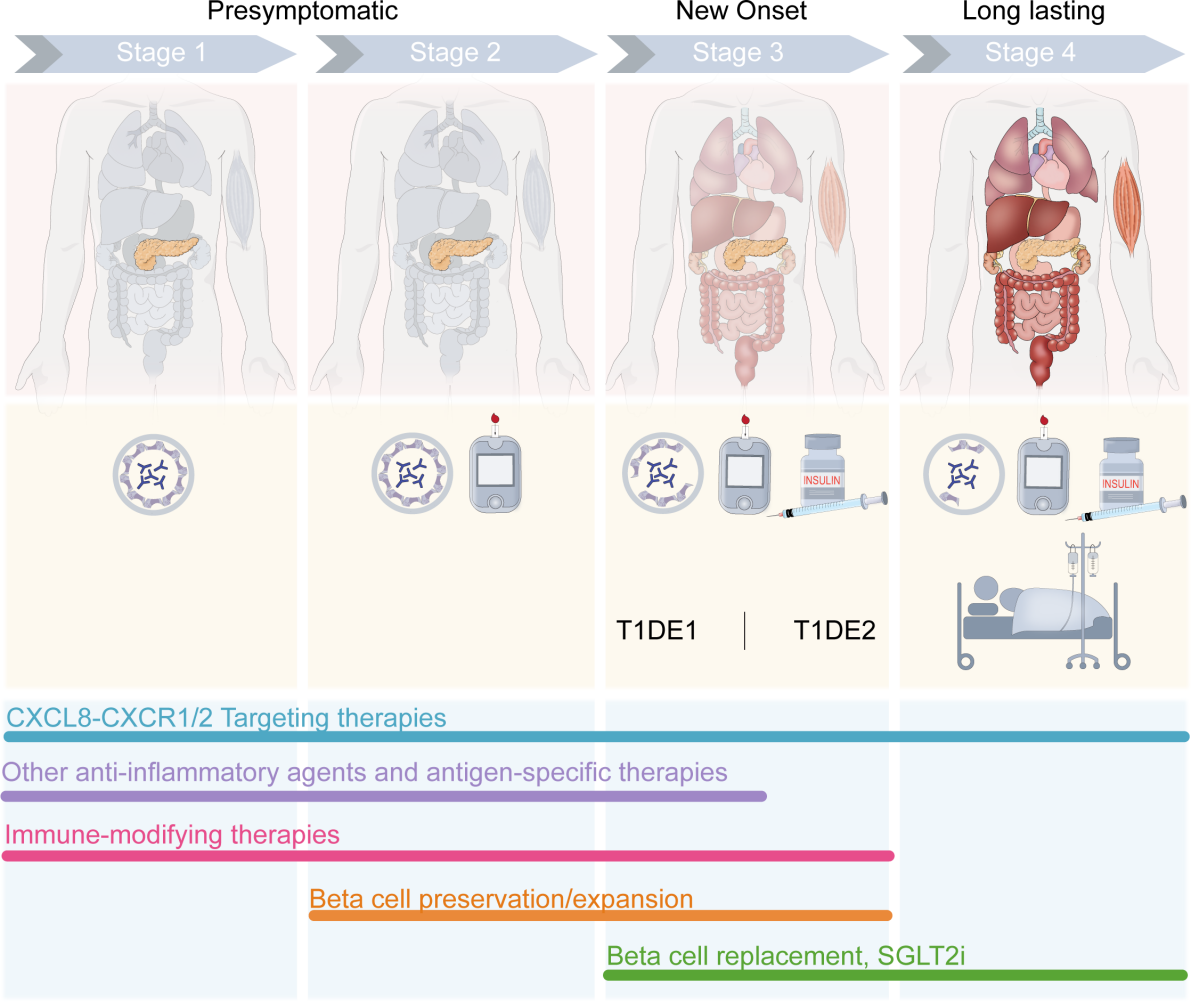
Stage-specific combination therapy strategies for T1D. CXCL8-targeting therapies show promise to improve outcomes across the stages of T1D, with evidence of CXCL8-CXCR1/2 axis involvement throughout the course of disease progression. In presymptomatic T1D, CXCR1/2 blockade may mitigate insulitis and β cell stress which could otherwise initiate autoimmunity. In new-onset T1D, CXCR1/2 blockade may mitigate or slow the progression of T cell migration to the pancreas and T cell-mediated autoimmune destruction of β cells. In late T1D, CXCR1/2 blockade may improve outcomes related to diabetic complications. When administered in combination with existing therapies developed for various stages of the disease, CXCL8-targeting therapies could improve outcomes. Anti-inflammatory agents, such as TNF- or JAK- inhibitors, and antigen-specific therapies (e.g., GAD-alum, oral insulin, multiple islet peptides) are developed for early presymptomatic T1D prior to the loss of β cells. Immunomodulating agents targeting T cells or B cells (e.g., anti-IL-21, anti-CD3, anti-CD80, anti-CD86, anti-CD20, and antithymocyte globulin [ATG]) and Treg-targeting agents (e.g., low-dose IL-2, Treg cell therapy) have shown promise to delay the onset or slow the progression of T1D through the development of Stage 3 T1D and are developed for Stage 1 up to Stage 3. Therapeutic strategies aiming to preserve or expand β cell mass/function (e.g., glucagon-like peptide 1 [GLP1] receptor agonists, calcium channel blockade [verapamil], monoamine oxidase A [MAOA] inhibition [harmine], and bone morphogenetic protein-7 [BMP-7]) are developed for Stage 2–3 T1D. For late-stage T1D, β cell replacement strategies and/or sodium-glucose transport protein 2 (SGLT2) inhibitors are being tested, with the aim to address pathologies driving severe diabetic complications associated with this stage of T1D.

Recognizing the three main stages of T1D—presymptomatic β-cell autoimmunity (stage 1), β-cell autoimmunity with dysglycemia (stage 2), and symptomatic disease onset (stage 3)—provides a framework for understanding disease progression and tailoring treatment strategies accordingly (**Figure 3**). The pivotal role of the axis in T1D pathogenesis suggests that its inhibition could complement existing treatment modalities to enhance the efficacy of current interventions, optimizing patient care and outcomes. Importantly, our understanding of the potential of CXCR1/2-targeting therapies and the key takeaways of the research summarized in this review is limited by the small number of trials conducted to date. Though promising, preclinical studies have not been able to address questions of efficacy specific to different endotypes and/or varying disease severity and progression. Most preclinical studies also rely on murine models, which lack a direct CXCL8 homolog, thereby limiting translational relevance. Moreover, the pleiotropic functions of CXCL8 in different tissues and cell types—beyond neutrophil chemotaxis—are incompletely characterized in the context of human autoimmunity. The temporal dynamics of CXCL8 expression in at-risk individuals, its role in epitope spreading, and its interaction with other inflammatory mediators require further investigation.

Current clinical trials targeting CXCR1/2 in T1D are limited in number and scope, emphasizing the need for additional studies assessing both immune outcomes and metabolic endpoints. Future research should prioritize the development of human-relevant models, biomarker-driven patient stratification, and combination therapeutic strategies. Elucidating these aspects will be critical to fully harness the therapeutic potential of CXCR1/2 blockade in T1D. Clinical trials to evaluate the safety and efficacy of CXCL8-CXCR1/2 axis inhibition, as a monotherapy or as an adjunctive therapy, in specific T1D populations (i.e., by endotype, baseline disease severity) represent a critical future research direction. Notably, the inclusion of analyses and/or additional trials to assess the potential for CXCR1/2-targeting therapies to address diabetic complications also represents a unique and impactful future direction of the field.

## 6 Conclusion

**Figure 4:**
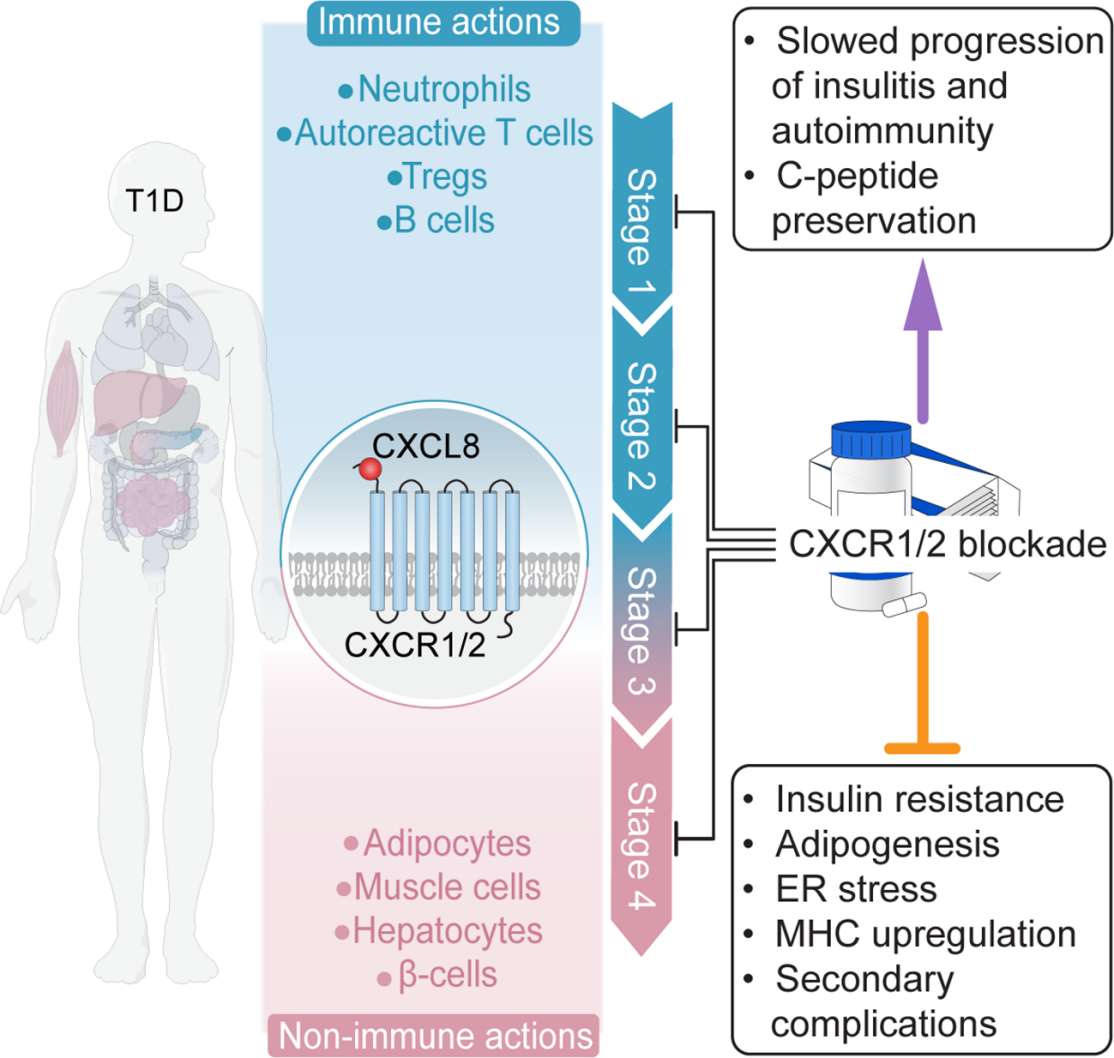
The potential for CXCR1/2-targeting disease-modifying therapies. The CXCL8-CXCR1/2 axis plays a crucial role in T1D onset and progression, acting through immune (e.g., neutrophils) and non-immune (e.g., β cells, adipocytes) pathways, such that CXCR1/2-targeting therapies represent a multifaceted treatment approach.

Long-term reliance on insulin is associated with growing safety concerns and socioeconomic costs. Disease-modifying therapies targeting the autoimmunity underlying T1D may be able to dramatically improve outcomes for patients and remain a highly sought-after clinical tool (**Figure 4**). The CXCL8-CXCR1/2 axis plays a crucial role in T1D onset and progression, acting through immune (e.g., neutrophils) and non-immune (e.g., β cells, adipocytes) pathways, such that CXCR1/2-targeting therapies represent a multifaceted treatment approach. Moreover, the considerable evidence supporting the involvement of the CXCL8-CXCR1/2 axis in secondary complications allows for speculations on direct beneficial effects of therapies targeting the axis also for common diabetic complications.

Importantly, CXCR1/2-targeting therapies show promise to capitalize on recent initiatives aiming to increase early screening for autoantibodies to identify patients with a high risk of T1D early (170, 171), when therapies can aim to preserve β cell function and delay or prevent symptomatic T1D onset and reliance on insulin. Strikingly, a recent study used machine learning with single-cell transcriptomics on pancreas tissues to detect unique gene signatures predictive of progression to T1D and specifically highlighted the importance of CXCL8 in this endeavor (170). In this study, across training instances, high cellular CXCL8 in the pancreas was consistently selected as a predictor of T1D.

In conclusion, the inhibition of the CXCL8-CXCR1/2 axis represents a promising therapeutic approach which, either as monotherapy or an adjunctive therapy, may lead to the prevention or reversal of T1D, with a meaningful potential clinical advantage conveyed by its role in multiple components of the disease pathology and its involvement in secondary diabetic complications.
